# Efficacy and Safety of Tetrabenazine in Reducing Chorea and Improving Motor Function in Individuals With Huntington's Disease: A Systematic Review

**DOI:** 10.7759/cureus.71476

**Published:** 2024-10-14

**Authors:** Nandini Vadlamani, Sabina Ibrahimli, Farees Ahmad Khan, Jason A Castillo, Kavya Sai Satya Amaravadi, Poornachandra Nalisetty, Safeera Khan

**Affiliations:** 1 Family Medicine, California Institute of Behavioral Neurosciences & Psychology, Fairfield, USA; 2 Internal Medicine, California Institute of Behavioral Neurosciences & Psychology, Fairfield, USA; 3 Medicine, California Institute of Behavioral Neurosciences & Psychology, Fairfield, USA; 4 Surgery, California Institute of Behavioral Neurosciences & Psychology, Fairfield, USA

**Keywords:** chorea, efficacy, huntington disease, motor function, tetrabenazine

## Abstract

Huntington's disease (HD) is a hereditary neurodegenerative disorder that causes chorea and motor dysfunction due to a mutation in the Huntingtin (HTT) gene. Tetrabenazine (TBZ) is used to treat HD-related chorea, but its efficacy and safety require further investigation. This systematic review aims to assess the efficacy and safety of TBZ in reducing chorea and improving motor function in HD patients. A comprehensive search was conducted across multiple sources, including PubMed, PubMed Central, Cochrane Library, Wiley Library, and Google Scholar. Medical subject heading (MeSH) terms were used to enhance search precision. Narrative reviews, clinical practice guidelines, open-label trials, and observational studies were included. Data synthesis followed Cochrane's recommendations for narrative synthesis. Evidence from narrative reviews, clinical guidelines, and trials consistently supports TBZ's efficacy in reducing chorea and improving motor function in HD patients. However, potential side effects like sedation and depression have been noted. This review underscores TBZ's positive impact but emphasizes cautious consideration of associated risks, informing clinical management and further research directions.

## Introduction and background

Also known as Huntington's chorea, Huntington's disease (HD) is a neurodegenerative hereditary disease that affects the brain and is caused by a mutation on the chromosome 4 Huntingtin (HTT) gene [1]. The mutation of the HTT gene is caused by cytosine-adenine-guanine (CAG) repeats expansion, which produces an elongated and mutated form of the HTT protein [2]. The mutated form of the HTT protein leads to the progressive degeneration of nerve cells in the brain, resulting in the various symptoms associated with the disease [3-6]. The most common symptoms of HD include motor manifestations, mainly chorea (involuntary motor movements), which affect more than 90% of HD patients [7]. These motor movements are usually jerky and unpredictable, affecting various parts of the body, which can slowly progress to rigidity and difficulty with coordination as the disease advances with time [8,9].

Various systematic reviews and meta-analyses have estimated the global prevalence of HD as 2.71 per 100,000 and its incidence as 0.38 to 0.48 per 100,000 per year [10,11]. Previous studies have also agreed that the prevalence and incidence of HD in the general population is the highest in Europe, North America, and Australia and lowest in Asia [10-12]. Although relatively rare, HD's manifestation of chorea can significantly impair the quality of life in patients with HD and their caregivers due to the caregiving burden [13]. Chorea can also increase anxiety and stigma in patients and their caregivers, especially those with high chorea [14]. Since HD does not currently have a cure [15,16], treatment modalities for reducing chorea are highly desirable as they can help improve the quality of life, mental health, and social outcomes of patients with HD as well as their caregivers.

Some treatment strategies commonly used for HD's chorea include tetrabenazine (TBZ), a vesicular monoamine transporter 2 (VMAT2) inhibitor [17]. The drug works by enhancing the regulation of dopamine release in the brain by inhibiting the activity of the VMAT2 protein [18]. VMAT2 is responsible for packaging neurotransmitters like dopamine into vesicles within the nerve cells and preparing them for release into the synapses [19]. Typically, chorea is caused by the imbalance of neurotransmitters like dopamine in the brain. Therefore, TBZ is theoretically considered an efficacious treatment modality for chorea in HD by inhibiting excessive dopamine release. Empirical studies have previously confirmed that VMAT2 inhibitors like TBZ, deutetrabenazine, and valbenazine can effectively reduce chorea in HD patients, but each of them has its potential side effects [20,21]. Although this empirical evidence is readily available, no systematic review has comprehensively pooled together findings from various studies investigating the effectiveness of TBZ in reducing chorea and improving motor function in individuals with HD.

This systematic review investigated TBZ's efficacy in reducing chorea and improving motor function in HD patients. The review answered the following research question: "What is the efficacy and safety of TBZ in reducing chorea and improving motor function in individuals with Huntington's disease?" The following objectives are addressed in this review: (a) to examine the safety and efficacy of TBZ in reducing chorea and improving motor function in HD patients, and (b) to investigate the quality of studies reporting the efficacy and safety of TBZ. Answering the research question and addressing the objectives will help inform clinical practice guidelines and future research directions, ultimately enhancing the management and quality of life of individuals with HD.

## Review

Methodology 

This systematic review was conducted as per the Preferred Reporting Items for Systematic Reviews and Meta-Analyses (PRISMA) guidelines [22].

Information Sources and Search Strategy

The following electronic platforms were searched: PubMed, PubMed Central (PMC), Cochrane Library, and Wiley Library. Additionally, Google Scholar, hand searching, and searching bibliographies of closely relevant scholarly materials supplemented the main sources of information. The keywords employed in the search included Huntington's disease, chorea, and TBZ. In addition to these keywords, the National Library of Medicine's medical subject heading (MeSH) terms were also searched on PubMed [23] as follows: (((("Huntington Disease/drug therapy"[Mesh]) OR ("Huntington Disease/prevention and control"[Mesh])) OR ("Huntington Disease/therapy"[Mesh])) AND ("Chorea/drug therapy"[Mesh]) OR ("Chorea/prevention and control"[Mesh]) OR ("Chorea/therapy"[Mesh] ) AND ("Tetrabenazine/adverse effects"[Mesh]) OR ("Tetrabenazine/therapeutic use"[Mesh]) OR ("Tetrabenazine/toxicity"[Mesh]). Table 1 below summarizes the search strategy and the number of records identified from each information source outlined above.

**Table 1:** Search strategy and results as per information source NA: Not applicable

Keywords/Search Strategy	Database Used	Number of Results
"Huntington Disease/drug therapy"[Mesh] OR "Huntington Disease/prevention and control"[Mesh] OR "Huntington Disease/therapy"[Mesh] AND "Chorea/drug therapy"[Mesh] OR "Chorea/prevention and control"[Mesh] OR "Chorea/therapy"[Mesh]) AND "Tetrabenazine/adverse effects"[Mesh] OR "Tetrabenazine/therapeutic use"[Mesh] OR "Tetrabenazine/toxicity"[Mesh])	PubMed MeSH database	400 papers
Huntington's Disease AND Chorea AND Tetrabenazine	PubMed	219
Huntington's Disease AND Chorea AND Tetrabenazine	PubMed Central	510
Huntington's Disease AND Chorea AND Tetrabenazine	Cochrane Library	48
Huntington's Disease and Chorea and Tetrabenazine	Wiley Library	37
Huntington's Disease and Chorea and Tetrabenazine	Google Scholar	28
NA	Hand searching and searching bibliographies	6
Total papers		1,248
Total papers after duplicates removed	201 duplicates found	1,047

Inclusion and Exclusion Criteria

We selected studies published between 2000 and 2023, written in English, and peer-reviewed. The studies also needed to focus on patients (humans) diagnosed with HD and experiencing chorea or motor function impairment, aged 18-70 years. Articles were included if they investigated the effectiveness or safety of TBZ, but they were excluded if they focused on other VMAT2 inhibitors like deutetrabenazine and valbenazine. Studies were also excluded if they utilized a sample of participants with other medical conditions (e.g., cardiovascular disease or other serious comorbidities), severe cognitive impairment, or pregnancy. Studies whose full texts were not available online, grey literature, and non-peer-reviewed pre-prints were also excluded.

Study Selection

All the identified studies were imported to EndNote (Clarivate Plc, London, United Kingdom) and then transferred to Microsoft Excel (Microsoft Corp., Redmond, United States) where duplicates were removed. Afterwards, all studies published before 2000 were excluded. Two independent reviewers screened the titles and abstracts of the remaining records for eligibility. Studies that met eligibility at this point were sought for retrieval online, whereby those whose full texts were unavailable were excluded. The shortlisted studies were then subjected to full-text screening by the same two independent reviewers. Articles that met the eligibility criteria were finally shortlisted in the list of selected studies. In case of any disagreements between the two independent reviewers, consensus was sought through collaborative discussions.

Study Quality Assessment

The studies whose full texts met the eligibility criteria were assessed for quality by two independent reviewers using tailored quality appraisal tools depending on the research design. Most of the studies were narrative reviews and hence their quality was assessed using the Scale for the Assessment of Narrative Reviews (SANRA) [24]; randomized controlled trials (RCTs) were assessed using the Cochrane Risk of Bias (RoB2) tool [25]; observational studies were assessed using the Newcastle-Ottawa (NOS) tool [26]; clinical practice guidelines were assessed using Appraisal of Guidelines for Research and Evaluation II (AGREE II) [27]. In case of disagreement between the independent quality assessors, a collaborative discussion was undertaken to achieve consensus.

Data Extraction

The following data points were extracted from the selected studies: study details (authors, publication year, study design, and setting/country if applicable), participants characteristics (inclusion and exclusion criteria), and TBZ-related data (efficacy and safety). Two independent reviewers undertook the data extraction process; discussions resolved disagreements.

Data Synthesis

The data synthesis followed the Cochrane Collaboration's recommendations of conducting a narrative synthesis [28]. First, studies of the same research design were grouped (e.g., RCTs separate from systematic and narrative reviews). Under each group, a narrative synthesis was conducted, which involved identifying similarities and differences between studies and acknowledging factors contributing to the same based on how each study was conducted, such as participant characteristics and the specific approaches undertaken in each study [28,29].

Results

Study Selection 

**Figure 1:**
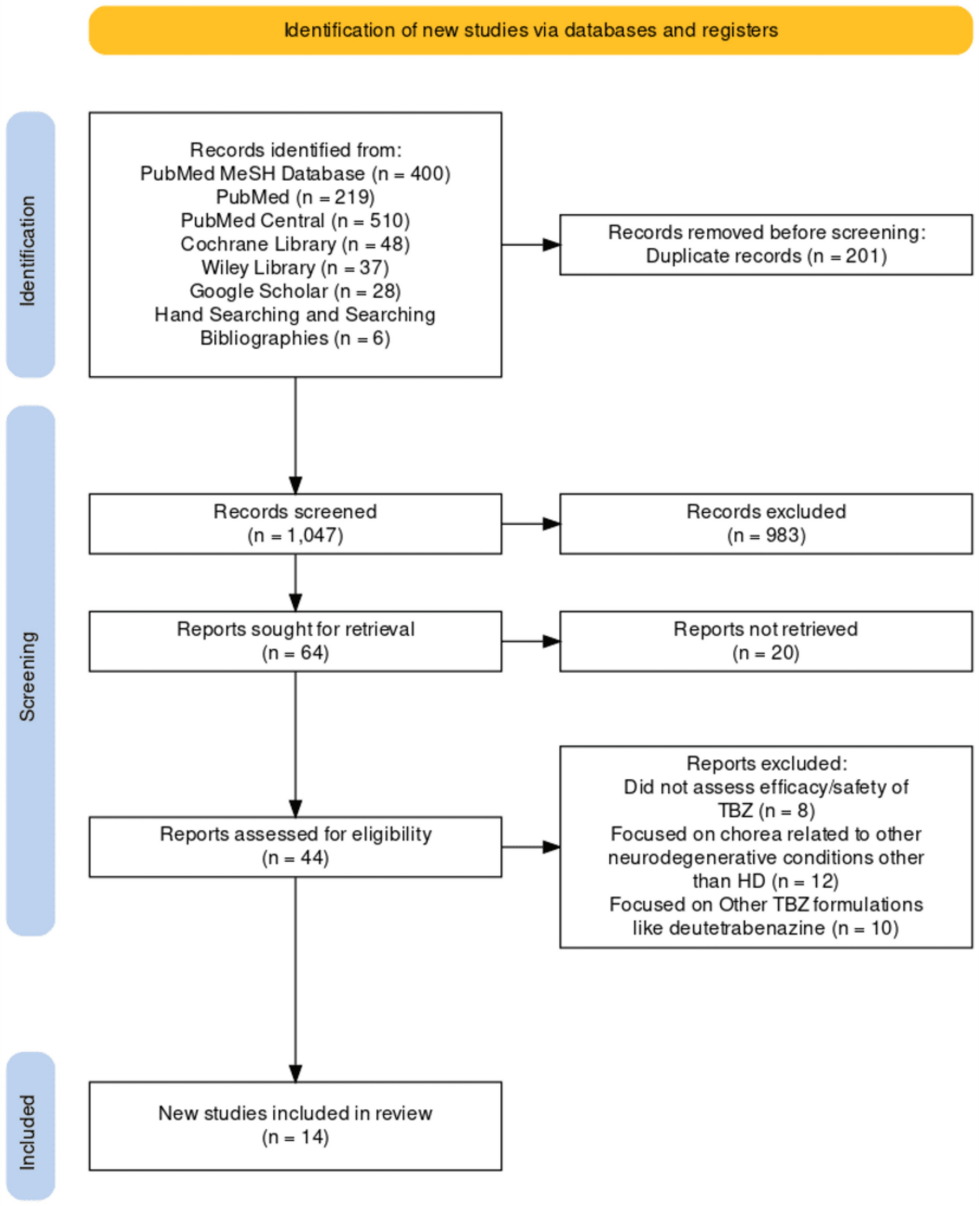
PRISMA flowchart PRISMA: Preferred Reporting Items for Systematic Reviews and Meta-Analyses; TBZ: Tetrabenazine; HD: Huntington's disease

We identified 1,248 records from all the information sources, of which 201 were excluded before screening for various reasons, such as being older than 2000 and published in non-English languages. Therefore, 1,047 records were screened for their titles and abstracts, of which 983 were eliminated. The remaining articles (n=64) were sought for retrieval, of which 20 did not have full texts available online. The remaining 44 articles were screened for eligibility using the inclusion and exclusion criteria for their full body texts; eight were excluded since they did not assess the efficacy or safety of TBZ, 12 were excluded because they focused on chorea related to other neurodegenerative diseases other than HD, and 10 were excluded because they focused on other TBZ formulations like deutetrabenazine without comparing their efficacy/safety with TBZ. As shown in Figure 1 below, fourteen articles eventually met the eligibility criteria and were selected for synthesis. This systematic review was done per the PRISMA guidelines [30].

Quality Appraisal Findings

The quality appraisal findings for narrative reviews are presented in Table 2. Due to the small number of other study designs, their quality appraisal findings are reported narratively.

**Table 2:** Quality appraisal findings for narrative reviews using SANRA SANRA: Scale for the Assessment of Narrative Review Articles

Study	Justification of Article Importance	Aims or Questions	Literature Search	Referencing	Scientific Reasoning	Data Presentation	Total Score (%)
Paleacu, 2007 [31]	0	2	1	2	2	2	9 (75%)
Yero and Rey, 2008 [32]	2	2	0	2	2	2	10 (83.33%)
Frank et al., 2008 [33]	2	2	0	2	2	2	10 (83.33%)
Fernandez et al., 2012 [34]	2	2	0	2	2	2	10 (83.33%)
Videnovic, 2013 [35]	2	0	0	2	2	2	8 (66.67%)
Dean et al., 2018 [36]	2	2	0	2	2	2	10 (83.33%)
De Tommaso et al., 2011 [37]	2	2	2	2	2	2	12 (100%)

Furthermore, the open-label RCTs by Frank (2009) were assessed for quality using Cochrane's RoB2 tool [38]. There was a high risk of bias because participants and researchers were aware of the treatment assignments, which introduced potential bias. However, the RCTs met other criteria of the RoB2 tool apart from the blinding concerns. The indirect comparison RCT by Claassen et al. (2017) was also assessed by the RoB2 tool, but the two RCTs from which data for indirect comparison were obtained were specifically assessed [39]. The two RCTs had minimal risk of bias since they were double-blind and met several RoB2 criteria without major concerns. Therefore, the two RCTs compared were high quality (the Huntington Study Group conducted them). They were very similar regarding design, baseline participant characteristics, and construct/outcome definitions. 

The quality of the clinical practice guideline (CPG) by Armstrong and Miyasaki was of moderate quality because it missed some of the important domains of the AGREE II instrument, mainly applicability and stakeholder involvement [40]. The authors did not identify the facilitators and barriers to implementing the CPG. Also, the CPG missed important aspects of the stakeholder involvement domain because the views of the intended users (HD patients with chorea) were not captured. However, the authors clearly and accurately established its scope, purpose, development rigor, presentation clarity, and editorial independence.

Finally, the three observational studies were appraised for their quality using NOS. Table 3 presents the quality assessment findings.

**Table 3:** Quality assessment of observational studies using NOS Newcastle-Ottawa quality assessment scale: Selection (****); Comparability (*); Outcome (***). A study can be awarded a maximum of one asterisk for each numbered item within the selection and exposure categories. A maximum of two asterisks can be given for comparability. NOS: Newcastle-Ottawa Scale

Study	Selection	Comparability	Outcome
Ferrara et al., 2012 [41]	****	*	**
Shen et al., 2013 [42]	***	**	**
Kegelmeyer et al., 2014 [43]	***	**	**

Study Characteristics

We reviewed 14 papers, of which seven were narrative reviews, two were open-label trials, one was an indirect comparison RCT, two were open-label prospective observational studies, and one was a retrospective observational study. All the studies involved HD patients manifesting with chorea and investigated the efficacy or effectiveness of TBZ. All the studies excluded participants who were on other treatment modalities for HD. The studies used various instruments to assess chorea and other motor function reductions. Table 4 summarizes the characteristics of the papers selected for review.

**Table 4:** Evidence summary table RCT: Randomized controlled trial; TMC: Total motor chorea; TBZ: Tetrabenazine; HD: Huntington's disease; CPG: Clinical practice guideline; NA: Not applicable

Citation	Study Design	Sample Size	Country	Participant Characteristics		Efficacy	Safety
				Inclusion	Exclusion		
Paleacu, 2007 [31]	Narrative review	NA	NA	HD patients with chorea	Not specified	All the studies reviewed report significant reductions in chorea; outcomes reported differently between studies.	Sedation; depressed mood; anxiety; insomnia; akathisia; one study reported one case of suicidal ideation.
Yero and Rey, 2008 [32]	Narrative review	NA	NA	HD patients with chorea	Not specified	All three studies reporting the efficacy of TBZ agreed on its effectiveness in reducing chorea in HD patients.	Akathisia; anxiety; depression; parkinsonism; suicidal ideation; fatigue; diarrhea.
Frank et al., 2008 [33]	Open-label RCT	30 patients	USA	Diagnosed with HD; have been on TBZ for at least two months; chorea clinically responsive to TBZ	Diagnosed with HD; have been on TBZ for at least 2 months; chorea clinically responsive to TBZ	After withdrawal from TBZ, TMC scores increased by 5.3 units from day 1 to 3.	No adverse effects after abrupt withdrawal.
Fernandez et al., 2012 [34]	Narrative review	NA	NA	HD patients with chorea	Not specified	Reviewed studies supported both the short- and long-term effectiveness of TBZ in reducing chorea in HD patients.	Sedation/drowsiness; depressed mood; anxiety; insomnia; akathisia; parkinsonism; dysphagia.
Videnovic , 2013 [35]	Narrative review	NA	NA	HD patients with chorea	Not specified	The authors reviewed two studies investigating the efficacy of TBZ in reducing chorea in HD patients; all two studies reported it as effective.	Depression; drowsiness; anxiety; parkinsonism; fatigue; akathisia; gastrointestinal distress; neuroleptic malignant syndrome.
Dean et al., 2018 [36]	Narrative review	NA	NA	HD patients with chorea	Not reported	TBZ has equivalent efficacy as deutetrabenazine.	Deutetrabenazine is safer than TBZ regarding adverse effects.
De Tommaso et all., 2011 [37]	Narrative review	Four studies with a total sample of 195 participants in the TBZ group and 30 participants on placebo	NA	HD patients with chorea	Not specified	All studies reported the TBZ as effective in reducing chorea in HD patients both in the short and long term; the effect slightly diminished over time (maximum observation time ranging between 12 weeks and 138 + 100 weeks).	Patients reported at least one side effect (not summarized in the review).
Frank, 2009 [38]	Open-label RCT	45 subjects	USA	Diagnosed with HD	On other treatment modalities for HD and chorea; participants on antidepressants, antianxiety, and other psychotropic medications but on stable doses were included	At week 80, the mean TMC score was reduced by 4.6 units.	Sedation; depressed; mood; anxiety; insomnia; akathisia.
Classen et al., 2017 [39]	Indirect treatment comparison utilizing data from two separate RCT	There were 174 patients, 90 from the first trial and 84 from the second trial	USA	Manifest of HD-related chorea; ambulatory	Disabling mental illness (e.g., depression and anxiety); dysphagia, dysarthria	Not assessed	Deutetrabenazine was associated with a significantly lower risk for adverse effects than TBZ, including agitation, akathisia, drowsiness, parkinsonism, and insomnia.
Armstrong and Miyasaki, 2012 [40]	CPG	NA	USA	HD patients with chorea	Not specified	The CPG recognized the efficacy of TBZ in reducing chorea in HD patients in previous research.	The CPG raised concerns about depression and Parkinson’s as TBZ side effects and advised always weighing benefits before prescribing the drug.
Ferrara et al., 2012 [41]	Open-label observational study	11 ambulatory patients	USA	Ambulatory patients with HD-related chorea; no prior pharmacological treatment for HD-related chorea within the past 30 days	Psychical and psychiatric symptoms that may hinder the participant from participating in the study	When patients are off-TBZ (either no prescription or after 24 hours washout), the mean maximal chorea score was 11.1, which reduced to 8.5 while on TBZ (statistically significant). However, no significant functional gains (e.g. cognitive aspects of HD with chorea suppression were recorded. Hence, supplemental therapy targeted at the cognitive aspects of HD is required.	Not assessed
Shen et al., 2013 [42]	Open-label observational study	98 patient	USA	HD patients with chorea that significantly disrupts activities of daily living; chorea not responsive to other treatment modalities	Unable to provide informed consent; unwilling to participate	75% of the HD patients with chorea demonstrated a significant reduction in chorea (moderate to large effect sizes).	86% of the patients reported at least one adverse effect; the most common ones include drowsiness, insomnia, depression, accidental injury, dysphagia, parkinsonism, weight loss, increased salivation, akathisia, nervousness, anxiety, asthenia, diarrhea, nausea, pain, constipation.
Kegelmeyer et all., 2014 [43]	Observational study	11 patients	USA	HD patients with chorea on a stable TBZ do for atleast 30 days	Significant improvement in motor score, mobility, balance, and sit-to-stand test when on TBZ compared to when not on TBZ	Comorbid neurological disorder; comorbid orthopedic condition; pregnancy inability to consent.	Not assessed
Frank, 2010 [44]	Narrative review	Four studies with a total sample of 195 participants in the TBZ group and 30 participants in placebo	NA	HD patients with chorea	Not specified	One study reported the short-term effectiveness of TBZ, and three studies reported the long-term effectiveness of TBZ in reducing chorea in HD patients.	Drowsiness; insomnia; agitation; depressed mood; fatigue; suicidal ideation; dizziness; parkinsonism; gastrointestinal distress; falls; dysphagia; dysarthria.

Discussion

Many researchers have focused on evaluating TBZ's efficacy and safety alongside its pharmacology and practical uses. In 2008, the United States US Government approved TBZ as a safe and effective pharmacological treatment for chorea related to HD [45]. One of the studies that provided evidence for its approval by the United States Food and Drugs Authority (FDA) included an RCT conducted by the Huntington Study Group and published in 2006 in Neurology [46]. The study involved 84 patients selected from 16 sites around the United States and established that TBZ is generally safe and tolerable for long-term use, leading to its approval by the FDA. This study has also remained a cornerstone source of evidence in most narrative reviews and clinical practice guidelines. All the narrative reviews and clinical practice guidelines included in this systematic review cited this study as their main source of evidence, supporting the efficacy and tolerability of TBZ for treating chorea in HD patients. The study was also frequently cited in the background sections of the open-label trials, indirect comparison RCTs, and observational studies selected for this systematic review, further highlighting its impact on the evidence supporting TBZ's efficacy and safety. Due to this reason, this study by the Huntington Study Group was not included among the studies reviewed as we aimed to review emerging evidence in the field, as per the recommendations provided by Gibson and Claassen [45] regarding the need for further evidence from observational and experimental studies assessing the long-term use of TBZ in treating HD-related chorea. However, extension studies utilizing participants from the original RCT by the Huntington Study Group, such as the open-label trial by Frank (2009), were considered for inclusion. Based on the latest evidence, the effectiveness and safety of TBZ are discussed below.

TBZ Efficacy

All the narrative reviews included in this systematic review supported the effectiveness of TBZ in reducing chorea in HD patients. A narrative review by Paleacu focused on studies published since the 1960s, noting that all of them supported the efficacy of TBZ. Although they did not extract crucial statistics from these studies, such as standard deviations and means, as well as confidence intervals/levels, later studies assessing the long-term efficacy of TBZ, including the RCT by the Huntington Study Group and four other observational studies, supported earlier studies regarding the efficacy of TBZ. Their findings are consistent with the narrative review conducted by Yero and Rey, which reviewed three studies supporting the efficacy of TBZ in reducing HD-related chorea, including the RCT conducted by the Huntington Study Group [46]. Yero and Rey presented the findings of the three studies separately, unlike the narrative synthesis conducted by Paleacu, where studies were integrative. Regardless of their approaches, none of the studies synthesized in both reviews provided contrary findings regarding the effectiveness of TBZ. The same trend was noted in the remaining narrative reviews, mainly because they cited the RCT by Huntington Study Group and a few other observational studies [34-37]. The clinical practice guideline published by Armstrong and Miyasaki also utilized the same approach as the narrative reviews to synthesize the literature, whereby they presented the same findings, arguing that TBZ has a large effect size in reducing chorea in HD patients and is relatively tolerable, which is the main reason they recommended it as the first-line treatment. The main source of evidence was the RCT by the Huntington Study Group, which was backed up by observational studies that have been published since then. Since the narrative reviews and the clinical practice guideline did not perform a meta-analysis, probably because of insufficient RCTs assessing its effectiveness in reducing chorea in HD patients, there is a need for larger clinical trials to provide sufficient evidence that can be analyzed using a meta-analytic synthesis.

Apart from the RCT conducted by the Huntington Study Group [46], open-label and indirect comparison trials have also been conducted to evaluate the efficacy of TBZ in reducing HD-related chorea. Before presenting the findings of these studies, it is imperative to discuss the RCT by the Huntington Study Group. The study involved randomizing HD patients to placebo (n=30) and TBZ (n=54) groups in an incremental dose of up to 100 mg/day for 12 weeks. The authors observed a 5.0-point reduction in chorea in the TBZ group, compared to a 1.5-point reduction in the placebo group, as assessed by the Unified Huntington Disease Rating Scale (UHDRS). Therefore, the study reported the short-term effectiveness of TBZ. The effectiveness of TBZ in the long term was confirmed by an open-label trial that utilized the participants of the Huntington Study Group and extended the follow-up period to up to 80 weeks with an incremental dose of up to 200 mg/day. Of the 75 participants invited, 45 completed the 80 weeks of follow-up, and there was a significant reduction in chorea to 4.6 points (SD=5.5) from baseline in the UHDRS. The findings are consistent with another open-label RCT that utilized a sample of 30 HD patients with chorea randomized into three groups: the withdrawal, partial withdrawal, and no-withdrawal groups. In the withdrawal group, chorea scores measured with UHDRS increased by 5.3 points from day 1 to day 3, and 3.0 points increase in chorea scores from day 1 to day 3 in the partial withdrawal group. Therefore, the three RCTs agreed that TBZ is an effective treatment modality for chorea in HD patients.

Observational studies further backed up findings from the narrative reviews and RCTs above regarding the effectiveness of TBZ. One observational study utilizing a sample of 11 HD patients with chorea exposed them to a period of withdrawal and a period of a stable TBZ dose. In the clinical practice guideline, the authors were particularly concerned with depression, suicidality, and parkinsonism, but they also reported other adverse effects (AEs), such as anxiety, sedation, and insomnia [40]. Chorea scores measured with UHDRS improved from 11.1 (SD=2.9) to 8.5 (SD=3.9) while on TBZ [41]. However, no significant functional gains were achieved as measured with Jebsen-Taylor Hand Function Test (JTHFT) and Berg Balance Scale. Their findings were backed up by another observational study that used retrospective data from 98 HD patients with chorea who had been on TBZ for a mean period of 3.1 years [42]. They found that 75% of the patients had either marked or very good responses to TBZ, pointing out the long-term efficacy of the treatment. In another observational study that utilized a sample of 11 patients, the authors assessed gait measures in forward walking, balance and mobility measures, and hand and forearm function measures while on and off TBZ. They found that mobility and balance scores, as measured by the UHDRS, improved significantly when on TBZ compared to when off TBZ, but gait measures and hand and forearm function did not improve. The findings are consistent with those of Ferrara et al., who also found no significant improvements in functional tests, which warrants further investigation in future observational studies and trials.

TBZ Safety

Various AEs associated with TBZ were reported in most articles. The narrative reviews reported various AEs that were reported in previous studies. Paleacu pointed out suicide from the studies as a concerning AE associated with TBZ. Yero and Rey reported a wide range of AEs but were particularly concerned with suicidality, depression, and neuroleptic malignant syndrome (NMS). Other AEs they reported as common were akathisia, parkinsonism, sedation, anxiety, and fatigue, all of which prompted dose adjustments. There was a consistent reporting of AEs associated with TBZ in all narrative reviews, including all the above, plus dizziness and insomnia [34-37]. AEs reported in RCTs are further discussed below.

Most of the AEs reported in the narrative reviews above were drawn from the RCT by the Huntington Study Group [46]. Additionally, in the long-term (80 weeks follow-up), the participants of the Huntington Study Group reported the following AEs: akathisia (20%), insomnia (22.22%), anxiety (28.89%), depressed mood (37.78%), and sedation (40%). When TBZ is suddenly withdrawn, HD patients may experience mild AEs, namely anxiety, decreased appetite, diarrhea, dysphagia, hallucinations, increased restlessness, tongue ulceration, mood swings, obsessive reaction, insomnia, and difficulty swallowing. Claassen conducted an indirect tolerability comparison between TBZ and deutetrabenazine utilizing data from two separate RCTs that were similar in design, and baseline participant characteristics were almost the same. The Huntington Study Group conducted the two RCTs, making it possible to perform an indirect tolerability comparison due to these similarities and minimal differences between the RCTs. They found that deutetrabenazine was associated with a significantly lower risk of AEs than TBZ, which include depression, agitation, akathisia, sedation, insomnia, and parkinsonism. They concluded that deutetrabenazine has a better tolerability profile compared to TBZ.

The same trend in AEs associated with TBZ was also noted in observational studies. Shen et al. (2013) [42] reported that the most common AEs reported by participants included dysphagia (19%), accidental injury (26%), depression (31%), insomnia (33%), and sedation (39%). The other two observational studies did not report AEs.

Limitations

Most of the articles were narrative reviews that synthesized observations from nearly the same previous primary studies, especially the Huntington Study Group RCT published in 2006 in Neurology. There were a few observational studies, whereby two of them utilized a small sample size and one a moderately large sample size. There were also a few RCTs, with two of them being open-label trials with potential bias.

## Conclusions

The purpose of this systematic review was to look at the effectiveness and safety of TBZ in decreasing chorea and increasing motor function in people with HD. The data was gathered from narrative reviews, clinical practice recommendations, open-label trials, indirect comparative studies, and observational studies for the review. The body of data consistently supports the efficacy of TBZ in decreasing chorea and increasing motor function in people with HD. The narrative reviews and clinical practice recommendations, as well as the Huntington Study Group's RCT, all emphasized the good effect of TBZ on chorea reduction. Long-term open-label studies showed TBZ's long-term efficacy across longer treatment durations. Observational studies revealed improvements in chorea scores while on TBZ, while functional benefits were not consistently seen. Although the research shows that TBZ is effective, it is vital to recognize the possible negative effects of its usage which include sedation, sadness, anxiety, and akathisia. This systematic review's results provide useful insights for doctors, researchers, and caregivers engaged in the treatment of HD patients.
